# Growth Restriction in Balb/c Mice Irradiated With X-Rays During Late Gestation: Role of Irradiation Timing, Dose Fractionation and Adaptive Response

**DOI:** 10.1177/15593258251395327

**Published:** 2025-12-07

**Authors:** Shayenthiran Sreetharan, Trista King, Christopher Thome, Neelam Khaper, Douglas R. Boreham, Sujeenthar Tharmalingam, Simon J. Lees, T.C. Tai

**Affiliations:** 1 Medical Sciences Division, 643660 NOSM University, Thunder Bay, ON, Canada; 2 Medical Sciences Division, 26627 NOSM University, Sudbury, ON, Canada; 3 Department of Physics and Astronomy, McMaster University, Hamilton, ON, Canada; 4 Department of Biology, 7890 Lakehead University, Thunder Bay, ON, Canada; 5 School of Natural Sciences, Laurentian University, Sudbury, ON, Canada; 6 Health Sciences North Research Institute, Sudbury, ON, Canada

**Keywords:** ionizing radiation, growth restriction, fetoplacental growth, prenatal, IUGR

## Abstract

**Objectives:**

Exposure of the developing fetus to high doses of ionizing radiation during prenatal development can result in growth restriction of the fetus, or a reduction in offspring growth. The developmental stage of the offspring at the time of irradiation is of interest, in order to characterize any potential periods of sensitivity for radiation-induced growth restriction effects. The goal of the present study was the development of a mouse model of radiation-induced growth restriction, following X-ray irradiation during late gestation.

**Methods:**

Pregnant BALB/cAnNCrl mice were irradiated with different irradiation conditions from gestational day (GD) 14-17. Treatments included an acute dose of 1.82 Gy X-ray irradiation on GD 14, 15 or 16. The effects of dose fractionation were also studied with one group receiving 0.455 Gy x 4 daily fractions from GD 14-17 (cumulative dose of 1.82 Gy). Another group also received a pre-treatment with 61 mGy X-ray irradiation on GD 14, 24 h prior to the 1.82 Gy on GD 15, to test for the possibility of a radiation-induced adaptive response.

**Results:**

Evidence for growth restriction was observed in all irradiation groups, with the greatest degree of growth restriction observed in the 1.82 Gy on GD 14 group. Evidence for growth restriction was based on a reduced gestational weight gain by pregnant dams and significant decrease in fetal weight and length measurements. Evidence for an adaptive response was not observed in the present study, as the combination group had similar outcomes to the group that only received the 1.82 Gy challenge irradiation dose.

**Conclusion:**

The establishment of a mouse model of radiation-induced growth restriction during late gestation will facilitate the ability for future work into determining the precise cellular and physiological effects on offspring, and the development of future countermeasures to protect against such adverse effects.

## Graphical abstract

**Graphical Abstract:**
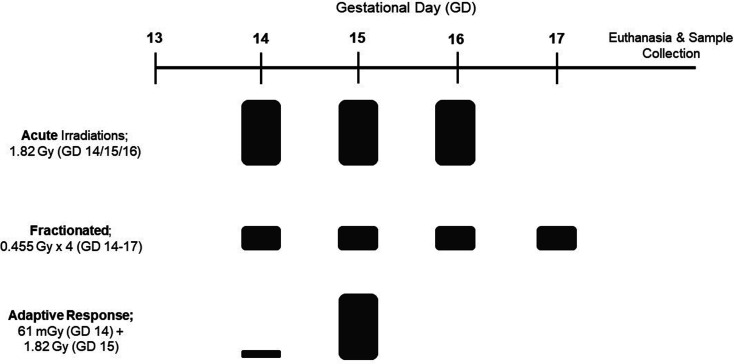

## Introduction

Exposure to high doses of ionizing radiation during prenatal development can result in a number of adverse outcomes to the developing fetus. This topic has been reviewed previously, with examples including De Santis et al. (2005),^1^ Groen et al. (2012)^2^ and Sreetharan et al^3^ (2017). The timing of irradiation during pregnancy can also influence the outcome, as there are changes in radiosensitivity of the developing conceptus at different stages of development. Prenatal radiation exposure results in growth restriction, which can be manifest as an irradiated offspring being born at a lower birth weight or reduced postnatal weight. Previous studies from our group have reported significant growth restriction in C57/Bl6J offspring irradiated with the highest tested dose of 1000 mGy of ^137^Cs gamma radiation on gestational day (GD) 15.^4,5^ This has also been reported in other studies at different gestational periods. For example, Kim et al. (2001)^6^ irradiated ICR mice with 2 Gy irradiation with gamma irradiation and reported significant decreases in offspring body length and weight in animals irradiated on GD 5.5, 7.5, 11.5 and 15.5. Similar effects are reported in later organogenesis and early fetal development periods. This includes the study by Minamisawa et al. (1990)^7^ that showed a dose-dependent decrease in body and brain weight in C57BL x C3H mice at doses of 1, 2 and 3 Gy on GD 14. A third example of growth restriction observed during later fetal development includes the study by Jensh et al^8^ (1995). The authors studied the effects of a 2 Gy X-ray irradiation on GD 17 in Wistar rats, and reported evidence for altered physiology in the irradiated offspring, based on permanent growth restriction, alterations in brain morphology and altered behavior. There is a need to better characterize radiation-induced growth restriction effects during this later fetal period of development (in the range of GD 14-18), as there is evidence for persistent growth restriction following irradiation in this developmental window, which is a potential area of concern for the health of the offspring.

Growth restriction effects are also observed with different types of ionizing radiation, such as neutrons and heavy ions. There have also been reports of prenatal radiation causing neurobehavioural effects in offspring, including a reduction in offspring brain weight. A study that highlights both of these features is an investigation by Ohmachi et al. (2004),^9^ in which B6C3F1 mice were irradiated with fast neutrons (0.1-1 Gy) or gamma rays (0.8 or 1.5 Gy) on GD 13.5. The authors observed whole body growth reduction at neutron doses as low as 0.5 and gamma irradiation of 1.5 Gy. The authors reported significant reduction in all measured tissue weights including the brain, thymus, heart, liver, spleen, lungs, kidneys and ovaries. The incidence and severity of the described effects are often dependent on irradiation conditions such as dose and gestational period of exposure. The effects also extend into altered behavioral and physiological outcomes in irradiated offspring, as reviewed in Sreetharan et al^3^ (2017).

The adaptive response (AR) has been demonstrated in a number of irradiation models ranging from cells to animals, with a review of prenatal AR studies provided by Streffer (2004).^10^ The radiation-induced AR involves the pre-treatment with a priming dose of radiation, which is typically a lower dose. The priming dose is followed by a larger, challenge dose of radiation. There is generally an attenuation in the response with the combination treatment when compared to the challenge dose alone. The radiation-induced AR during prenatal development has previously been studied in mouse models. For example, Boreham et al. (2006)^11^ was able to demonstrate an AR for teratogenic effects during organogenesis. Pregnant mice with varying genotypes of the *Trp53* gene (which encodes the p53 tumor suppressor protein) were irradiated with a combination of 0.3 Gy given 24 h before a challenge dose of 4 Gy on GD 11. The authors reported a significant protective effect of the priming dose, based on significantly less fetal tail shortening in the combination treatment group, compared to 4 Gy only group. A summary of mouse or rat prenatal AR studies is provided in Table 1. Adaptive response during the prenatal period in non-mammalian models have also been investigated, which includes zebrafish embryos^12^ and chicken embryos.^13^ One noteworthy study by Thome et al. (2017)^14^ demonstrated an adaptive response in lake whitefish embryos. The authors reported that a heat shock delivered 6 h prior to irradiation resulted in a 25% reduction in embryonic mortality. This study highlights the ability for other priming treatments, such as a mild heat shock, to induce an AR when combined with a challenge dose of radiation.

**Table 1.** Summary of Prenatal Radiation Adaptive Response Studies in Mouse and Rat Models

Citation	Model	Irradiation type	Priming dose + challenge dose (Gy)	Dose-rate (Gy min^-1^)	Irradiation days (gestational day)	Endpoints
Boreham et al.^11^	C57Bl/6-*Tp53* heterozygous mouse	^60^Co	0.30 + 4	0.51	10 + 11	Fetal tail length
					11 + 12	
Hays et al.^15^	Sprague-dawley rat	^137^Cs	0.02 + 5	0.4	14 + 15	Morphological assessments of the developing cerebral cortex
					(Δ1, 3, 6, 12 or 24 h)	
Howell et al. (2012)^16^	BALB/c mouse	Chernobyl-contaminated soil: ^137^Cs, ^90^Sr (priming)	100-130 mSv +2.4 Sv	10-13 mSv per day (priming)	F_0_: 7-16 (priming)	Micronucleus frequency
		^137^Cs (challenge)			F_1_: 16-18 days (challenge)	White blood cell levels
						Gene expression
Inouye et al. (1997)^17^	Slc: ICR mouse	X-ray	0.05 + 0.5		8 (Δ4 hours)	Neural tube defect frequency
			0.05 + 4			
Lee et al.^18^	ICR mouse	^60^Co (priming)	0.3 + 0.8	0.094 (priming)	10.5 + 11.5	Fetal size and weight
		Neutron (challenge)		0.9 (challenge)		Fetal malformations
						Fetal tail length
Mitchel et al. (2002)^19^	*Trp53*^+/+^, *Trp53*^+/−^, *Trp53*^−/−^ mouse	^60^Co	(Heat shock) + 4	0.0085	11 or 12 (Δ1 h)	Teratogenic effects of fetal tail and limb digits
Müller et al. (1992)^20^	Heiligenberger mouse	240 kVp X-ray	0.03/0.05/0.1 + 2/3/4/5/6	0.12 (low dose)	Various	Cell number
				1 (high dose)		Post-implantation development
Okazaki et al. (2005)^21^	ICR mouse	^137^Cs	0.02 + 2	0.000667 (priming)	9.5 (Δ4 h)	Apoptotic cell frequency
				1.04 (challenge)		Fetal malformations
						Fetal weight
Sreetharan et al.^4^	BALB/c mouse	320 kVp X-ray	0.061 + 1.82	0.222 (priming)	14 + 15	Fetoplacental weight and length
				0.838 (challenge)		
Wang et al. (1998; 1999; 2000; 2004a; 2004b; 2012)^22-27^	ICR mouse	200 kVp X-ray	Various	Various	Various	Fetal death
						Fetal malformations
						Fetal weight
						Apoptosis status
						*Trp53* gene status
Wang et al. (2022)^28^	C57BL/6J mouse	200 kVp X-ray (priming & challenge)	0.05/0.30 (X-ray) + 3.5	0.300 (X-ray, priming)	11 + 12	Fetal death
		C/Si/Fe ions (priming)	0.010 – 0.320 (ion) + 3.5	0.55 (X-ray, challenge)		Fetal malformations
				0.010-0.100 (ions)		Fetal weight
Wojcik et al. (1992)^29^	Heiligenberger mouse	240 kVp X-ray	0.05 + 2	0.12 (priming)	50 h post-conception +56 h post-conception	Chromosomal aberrations
				1 (challenge)		

The goal of the present study was the development of a radiation-induced growth restriction model using BALB/c mice. The effects of an acute, high dose of X-ray irradiation was studied at different gestational timepoints during late gestation via fetoplacental growth restriction measurements. The role of dose fractionation and the possibility of a radiation-induced AR was also investigated.

## Materials & Methods

### Animals

Male and female wild type BALB/cAnNCrl mice (7-8 weeks of age) were purchased from Charles River Laboratory (Montreal, QC, Canada) and used for breeding in this study. Animals were housed at the Lakehead University Animal Care Facility and all described protocols were approved by the Lakehead University Animal Care Committee (AUP# 1467646) in December, 2019. The study period was from May (purchase of animals and start of acclimation period prior to breeding) to November, 2021. Animals were maintained on a 12:12 light-dark cycle with 40%-70% room humidity. Food was available to animals *ad libitum*, with free access to water. The only exception was the removal of food and water from the animal cage during irradiations. Female breeder mice were fed the ProLab RMH 2000 breeding diet (content of protein = 18%, fat = 9% and fiber = 4%). Male mice were fed a standard lab chow diet for the duration of the study.

Breeding was performed by placing 2 female mice in a male cage and allowed to breed overnight. The following morning, females were removed, checked for vaginal plugs and placed in a new cage with additional cage enrichment materials. The morning following overnight breeding was designated as gestational day (GD) 0. Female breeder mice were housed singly for the remainder of the study. Pregnancy was confirmed based on gestational weight gain to day 13 prior to assignment to a treatment group. Pregnant dams were weighed daily beginning on gestational day 13.

Pregnant animals were euthanized via cervical dislocation following isofluorane on gestational day 18 and fetoplacental units were collected. Placenta and fetal samples were weighed and two morphometric measurements of the fetal were collected, crown-rump length and biparietal diameter. Following weight and length measurements, the fetal tail, placenta and whole fetus was flash frozen on dry ice and stored at −80°C until further processing.

### Irradiation and Dosimetry

Animal irradiations were performed using the XRAD320 cabinet X-ray irradiator system (Precision X-Ray; Madison, CT, USA). On the day of irradiation, food and water were removed from the cage, and the animal carefully transported via lab cart to the irradiator, which was also located in the animal facility. Sham-irradiated animals were similarly transported to the irradiator and placed inside for 5 min, with the source turned off. Sham-irradiated animals were transported daily between gestational days 14-17, in order to match the most frequently transported fractionated irradiation condition. A second group of animals (“Control”) also served as a control group, however this cohort of animals were never removed from the breeding room. This group was used to determine if the sham irradiation protocol of transporting animals to the irradiator resulted in any significant changes.

**Figure 1.**
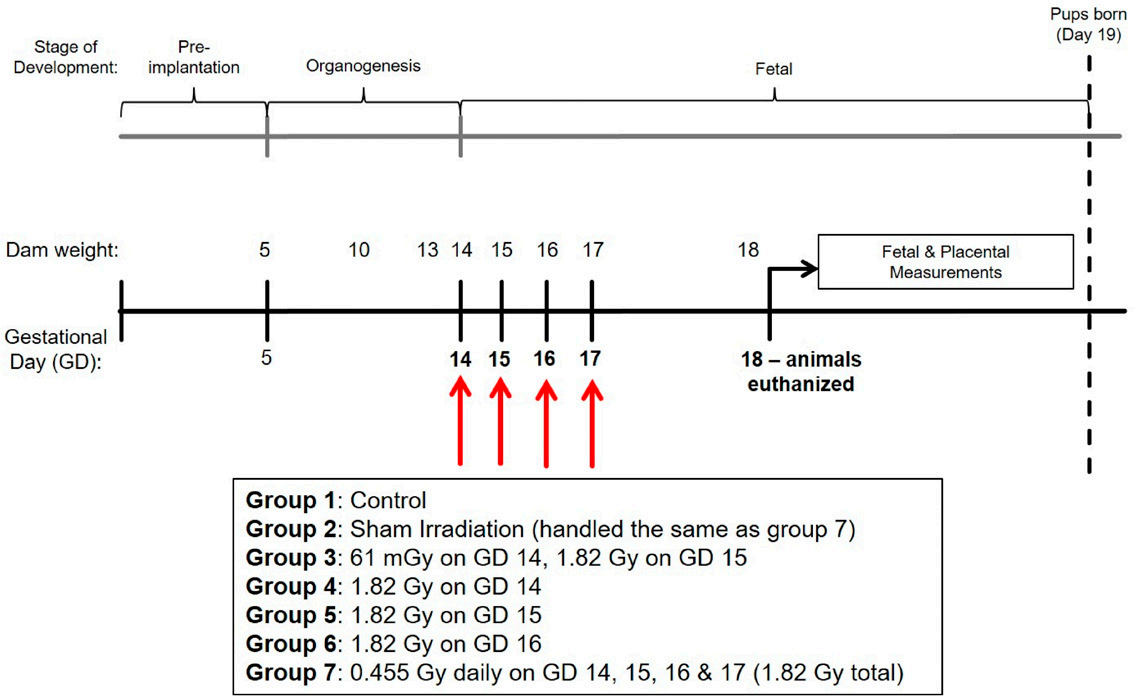
Summary diagram of experimental design and irradiation treatment conditions

A total of 5 different irradiation treatments were performed, with animals irradiated on single or multiple days between GD 14 to 17 of pregnancy. Three groups were irradiated with a single, acute dose of 1.82 Gy x-rays on GD 14, 15 or 16. A fourth group was irradiated in a fractionated schedule, with 4 × 0.455 Gy fractions performed on GD 14, 15, 16 and 17 (24 h between fractions and a total cumulative dose of 1.82 Gy). A fifth group was irradiated with 61 mGy x-rays on GD 14, 24 h prior to receiving an acute challenge dose of 1.82 Gy on GD 15. A summary of the irradiation treatment groups in this study are provided in Figure 1.

Dosimetry was performed using thermoluminescent dosimeters (TLDs; Mirion Technologies; Oak Ridge, TN, USA) with aged, male mice, due to a similar body composition and body weight (∼30 g) to the pregnant mice that were irradiated during late gestation. The aged mice were euthanized (n = 3 mice), placed in an empty animal cage with the food and water removed in order to simulate the irradiation conditions of the test animals. A TLD was placed below the ventral side of the animal in order to estimate an “exit” dose. The X-ray tube generates photons above the animal, therefore the ventral surface would approximate the attenuation of the photons as it passed through the body of the animal. This location was selected due to the proximity to the location of the developing fetus in a pregnant animal.

A total of 3 aged mice were used and the average readout between the replicates was reported as the absorbed dose-rate. From the calculated average dose-rate, the absorbed dose for each condition (2 Gy, 0.5 Gy and 100 mGy nominal doses) was calculated based on the exposure time of the animal. Two different irradiation program conditions were used in the study in order to accommodate for the difference in dose range in this study. Dosimetry was performed as described above for each irradiation program (using the same three test animals).

The results of the described dosimetry are summarized in Table 2. When performing irradiations, the order that the animals (with the implanted TLDs) were irradiated was randomized. For the irradiations in the present study, reported doses were verified using thermoluminescent dosimeters (Mirion Technologies GDS Inc; Oak Ridge, TN, USA) for the two different irradiation programs used in this study. 1.82 Gy and 0.455 Gy irradiations were performed at the following conditions: 320 kVp, 12.5 mA, predicted dose-rate = 0.921 Gy per minute, TLD verified dose-rate = 0.838 Gy per minute. The 61 mGy irradiations were performed at the following conditions: 320 kVp, 12.5 mA, predicted dose-rate = 0.364 Gy per minute, TLD verified dose-rate = 0.222 Gy per minute. These dosimetry results indicate that the actual absorbed dose for nominal doses of 2 Gy, 0.5 Gy and 0.1 Gy irradiations were 1.82, 0.455 and 0.061 Gy respectively. For the remainder of the paper, dosimetry verified absorbed doses are reported.

**Table 2.** Dosimetry Results From Thermoluminescent Dosimeters (TLDs) Placed on the Ventral Surface of Euthanized Animals to Estimate Fetal Dose

Irradiation program	Mouse	Estimated dose (cGy)	Measured dose from TLD (cGy)	Exposure duration (s)
High dose-rate	Mouse A	301.29	279.5	198
High dose-rate	Mouse B	301.2	272.7	198
High dose-rate	Mouse C	301.27	277.7	198
Low dose-rate	Mouse A	200.37	140.7	329
Low dose-rate	Mouse B	200.47	137.8	330
Low dose-rate	Mouse C	200.49	87.3	331

### Statistical Testing

GraphPad PRISM (GraphPad Software; San Diego, CA, USA) software was used for preparation of figures and statistical testing of animal data. Fetoplacental measures and litter sizes were compared across treatment groups using a 1-way Analysis of Variance (ANOVA), followed by either Dunnet’s multiple comparison post-hoc test (acute and fractionated effects) or Tukey’s multiple pairwise comparisons (adaptive response effects) post-hoc test if a significant result was detected for the ANOVA. Comparison of non-viable fetus frequency between treatments was performed using the chi-square statistic. Comparison of the study parameters between Control and sham-irradiated groups (to assess the effect of the sham-irradiation protocol) was completed using an independent sample *t*-test.

## Results

### Sham-Irradiation Protocol Effects

The effects in the sham-irradiated group were compared to Control group animals, in order to assess the possibility of the sham-irradiation protocol inadvertently impacting fetoplacental growth, apart from radiation exposure. Control animals were not removed from the animal breeding room for the duration of the study, and were not transported to the irradiator, as the sham-irradiated animals were. A comparison of study parameters between these two groups are presented in Table 3. The only statistically significantly different outcome measure was placenta growth, with a small, but significant decrease in placental weight in the sham-irradiated group (*t*-test, *P* = .035). For all other tested parameters, there was no significant difference between the two groups, suggesting a minimal involvement of the transportation and sham-irradiation of animals in the observed fetoplacental growth outcomes. Moving forward, all comparisons of irradiation treated animals will be made to the sham-irradiation cohort.

**Table 3.** Summary of Sham-Irradiation Protocol Effects on Study Measures. Comparison was Performed Between the Control Cohort of Dams, which Were not Removed From the Animal Breeding Area for the Duration of Gestation. Sham-Irradiated Dams Were Transported Daily to the Irradiator and Placed Inside, Without Exposing the Animals to X-Rays. Animals Were Transported at the Same Frequency of the Fractionated Treatment Group, which was the Most Transported Group. Sample Size (*N*) of Dams and Offspring is provided for Each Group. Independent Sample *t*-test was Completed to Compare Study Parameters of Fetal Weight (g), Crown-Rump Length (cm), Biparietal Diameter (cm), Placental Weight (g) and Fetoplacental Ratio, With Significant Pairwise Differences (*P* < 0.05) for Each Respective Study Measure Indicated With Bolded Text

	Control	Sham	*P*-value	Test statistic (d.f.)
*N*_dam_	10	9		
*N*_offspring_	55	86		
Fetal weight (g)	1.00 (0.1)	0.98 (0.1)	.52	t (139) = 0.6481
Crown-rump length (cm)	2.00 (0.2)	2.01 (0.1)	.76	t (138) = 0.3072
Biparietal diameter (cm)	0.66 (0.05)	0.67 (0.04)	.27	t (138) = 1.110
Placental weight (g)	0.11 (0.02)	0.096 (0.01)	**.035** ^a^	t (138) = 2.132
Fetoplacental ratio	9.99 (1.7)	10.37 (1.6)	.18	t (138) = 1.337

^a^Values are reported as mean (SD), and the test statistic and degrees of freedom (d.f.) are included for each study parameter.

### Maternal Weight Gain & Litter Statistics

**Figure 2.**
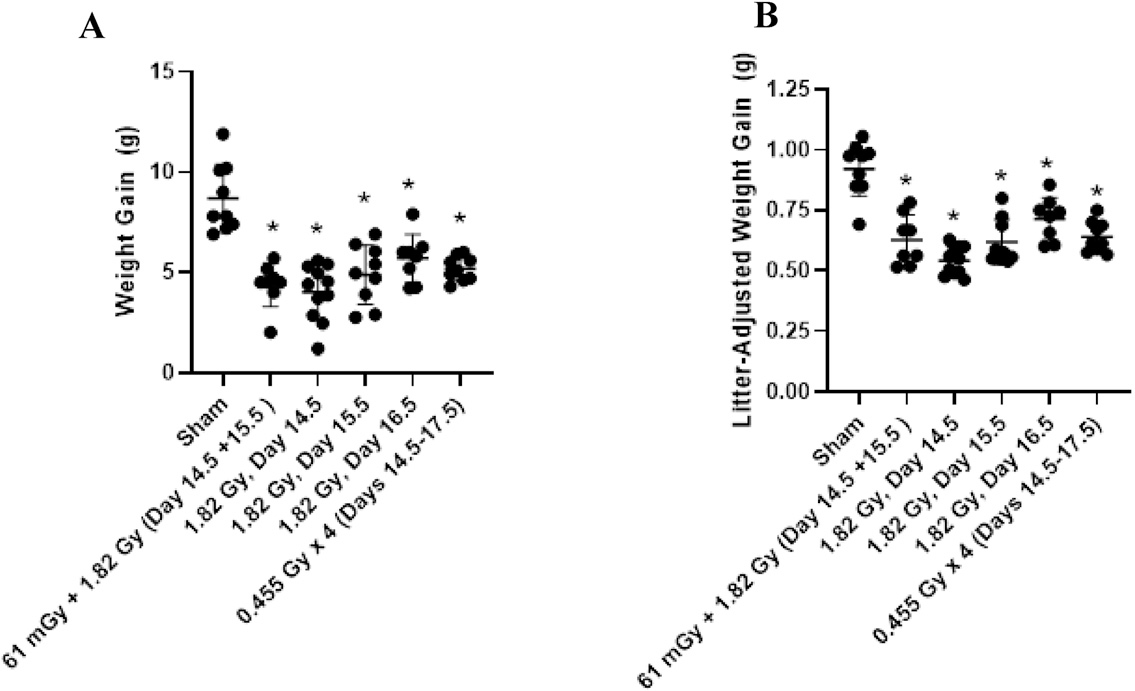
Maternal gestational weight gain following irradiations between gestational days 14 and 18. Weight gain is presented as both (A) Total weight gain or (B) Weight gain corrected for the dam’s litter size. Individual weight gain values for each dam are included for each group in both panels. * Denotes statistical significance to the sham irradiation group (1-way ANOVA; *P* < .05). Sample sizes (n) for treatment groups were: sham (n = 9), 61 mGy +1.82 Gy (n = 8), 1.82 Gy day 14.5 (n = 11), 1.82 Gy day 15.5 (n = 9), 1.82 Gy day 16.5 (n = 8), 0.455 Gy x 4 (n = 9)

Maternal weight gain between GD 14 and 18 are presented in Figure 2. Significant differences were observed between all treatment groups compared to the sham-irradiation controls, when considering the total weight gain (Figure 2A) or when corrected for the litter size (Figure 2B). Litter statistics of each treatment group are presented in Table 4. Litter size was not statistically significant between the treatment groups (1-way ANOVA; F_6,57_ = 1.676, *P* = .14). The number of non-viable fetuses for each treatment group is also included in Table 4. The frequency of non-viable fetuses was compared across the treatment group using the chi-square test, with no significant differences observed in the non-viable offspring frequency (χ^2^ = 7.667; *P* = .2636).

**Table 4.** Litter Outcomes of Sample Size, Total Number of Offspring, Average Litter Size and Total Number of Non-viable Fetuses are Reported for Each Treatment Group

Treatment	Number of dams (n)	Number of offspring (n)	Litter size (n ± SD)	Number of non-viable fetuses (% total)
Control	10	55	6.5 ± 4.6	0 (0 %)
Sham	9	86	10.17 ± 2.86	3 (3.5%)
61 mGy (day 14) + 1.82 Gy (day 15)	8	55	7.4 ± 3.0	2 (3.6%)
1.82 Gy, day 14	11	72	7.8 ± 1.64	1 (1.4%)
1.82 Gy, day 15	9	72	9.6 ± 1.95	1 (1.4%)
1.82 Gy, day 16	8	64	8.4 ± 2.07	2 (3.1%)
0.455 Gy x 4 (days 14, 15, 16, 17)	9	73	8.5 ± 0.58	1 (1.4%)

### Fetoplacental Measurements

Pregnant dams were euthanized via cervical dislocation following isofluorane administration on GD 18 and fetoplacental units were collected from all animals. For all fetoplacental measurements, a statistically significant difference was not detected between Control and sham-irradiated group (1-way ANOVA with Tukey’s post-hoc, *P* > .05). Moving forward, the sham-irradiated group will represent the reference Control group for statistical significance comparisons.

**Figure 3.**
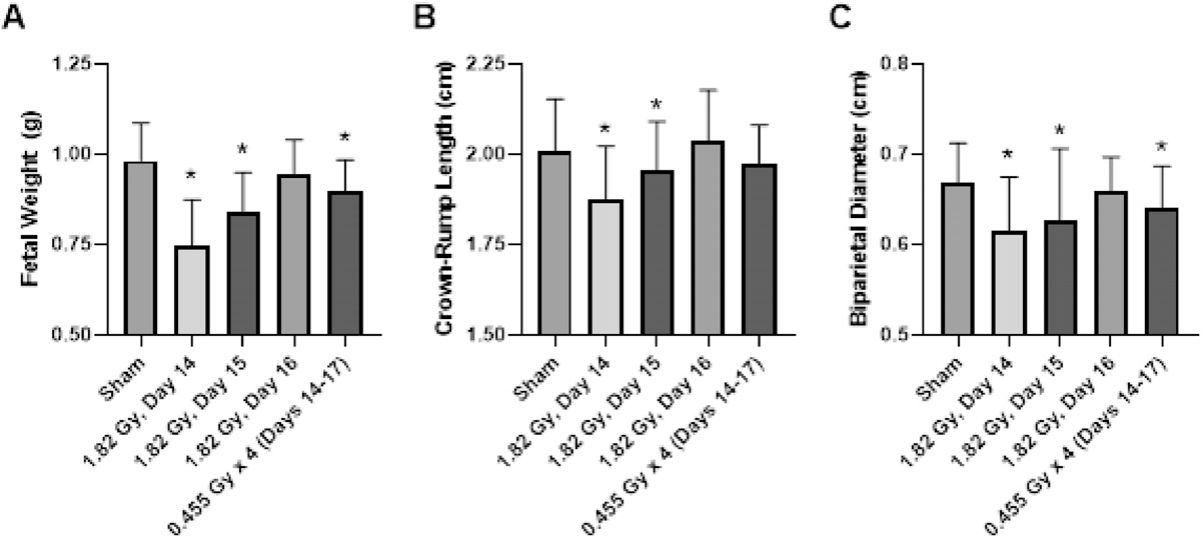
Effects of acute and fractionated irradiation during late gestation on fetal parameters. Fetal measurements of (A) Fetal weight, (B) Crown-Rump length, and (C) Biparietal diameter in gestational day 18 offspring treated with a single 1.82 Gy irradiation (on day 14, 15 or 16) or with a fractionated irradiation schedule (0.455 Gy x 4 Fractions). * Denotes statistical significance to the sham-irradiation control group (1-way ANOVA; *P* < .05). Error bars indicate standard deviation. Sample sizes (n) for treatment groups were: sham (n = 86), 1.82 Gy day 14.5 (n = 72), 1.82 Gy day 15.5 (n = 72), 1.82 Gy day 16.5 (n = 64), 0.455 Gy x 4 (n = 73)

When considering fetal measurements, the greatest degree of growth restriction was observed in the 1.82 Gy (day 14) group, and was statistically significant for all three fetal measurements of fetal weight, crown-rump length and biparietal diameter (Figure 3). The fractionated group also demonstrated evidence for growth restriction based on based on statistical significance in two of the three fetal measurements relative to sham-irradiated controls. There was minimal evidence for any influence of the 61 mGy pre-treatment in the combination group (61 mGy +1.82 Gy; day 14 + 15), as there was no statistical significance compared to the 1.82 Gy (day 15) for conceptus weight and crown-rump length fetal measurements (Figure 5) and both placental measurements (Figure 6) when considering the adaptive response treatment groups.

**Figure 4.**
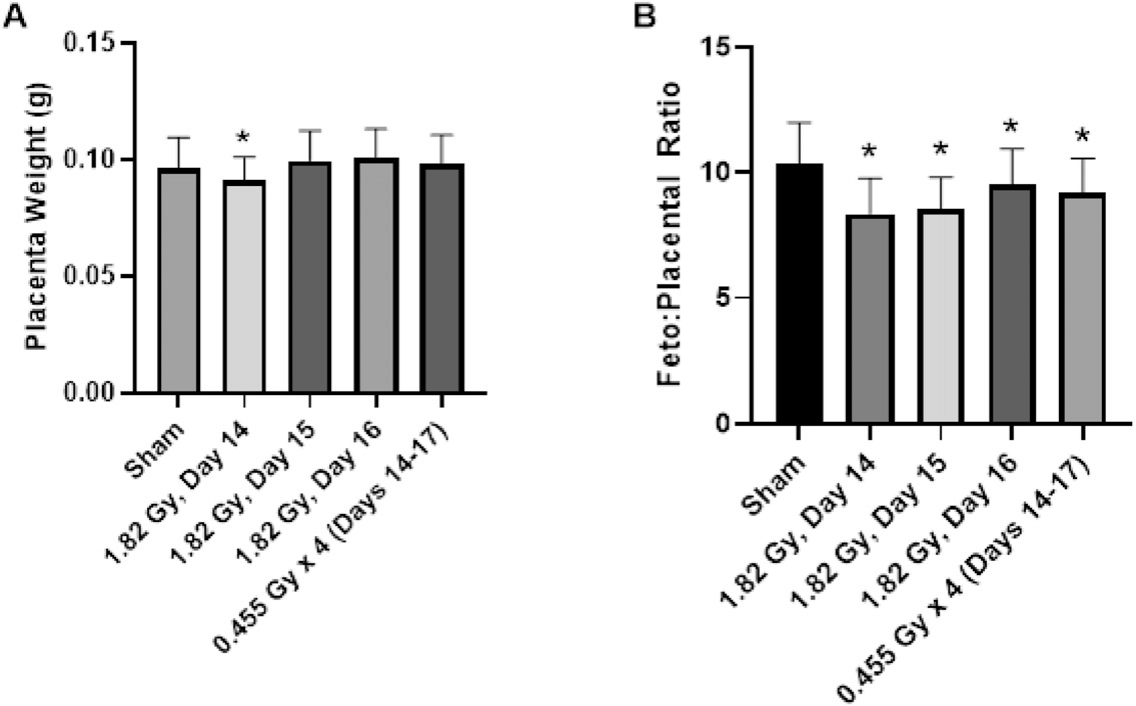
Effects of acute and fractionated irradiation during late gestation on placental parameters placental measurements of (A) Placenta weight and (B) Fetoplacental ratio in gestational day 18 offspring treated with a single 1.82 Gy irradiation (on day 14, 15 or 16) or with a fractionated irradiation schedule (0.455 Gy x 4 fractions). * Denotes statistical significance to the sham irradiation control group (1-way ANOVA; *P* < .05). Error bars indicate standard deviation. Sample sizes (n) for treatment groups were: sham (n = 86), 1.82 Gy day 14.5 (n = 72), 1.82 Gy day 15.5 (n = 72), 1.82 Gy day 16.5 (n = 64), 0.455 Gy x 4 (n = 73)

**Figure 5.**
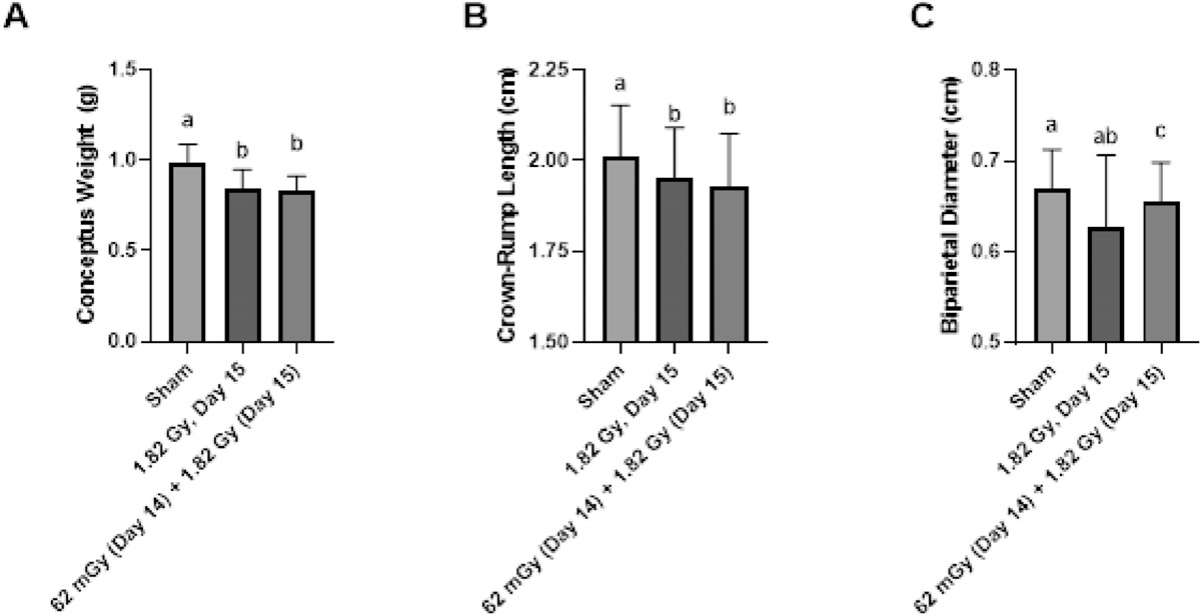
Fetal measurements in the testing of an adaptive response. Animals were irradiated with 1.82 Gy on day 15, in addition to a combination treatment of 62 mGy on day 14 + 1.82 Gy on day 15. Treatments that share unique letters above the error bar were Statistically significant pairwise comparisons. Sample sizes (n) for treatment groups were: sham (n = 86), 1.82 Gy day 15 (n = 72), 61 mGy +1.82 Gy (n = 55)

**Figure 6.**
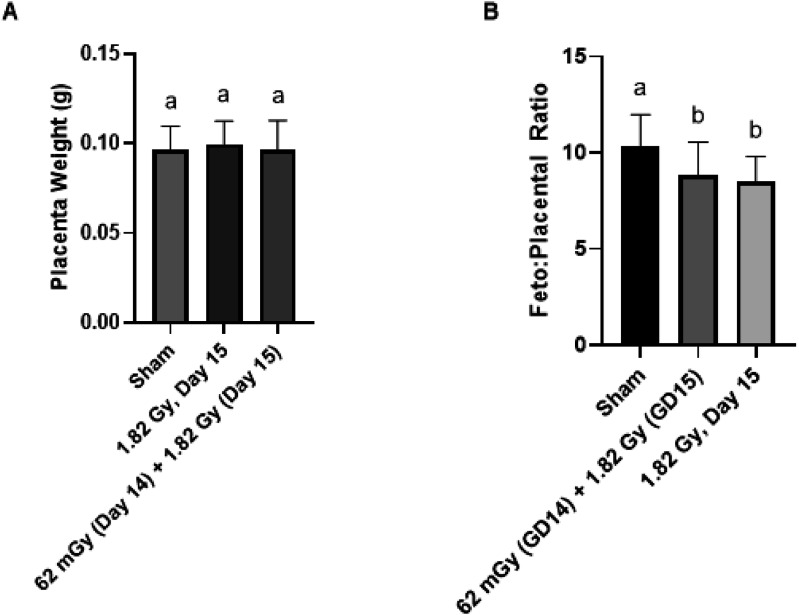
Placental measurements in the testing of an adaptive response. Animals were irradiated With 1.82 Gy on day 15, in addition to a combination treatment of 62 mGy on day 14 + 1.82 Gy on day 15. Treatments that share unique letters above the error bar were statistically significant pairwise comparisons. Sample sizes (n) for treatment groups were: sham (n = 86), 1.82 Gy day 15 (n = 72), 61 mGy +1.82 Gy (n = 55)

The placental weight was unchanged across treatment groups with no statistical significance detected between irradiated and sham-irradiated controls (Figures 4–6). Fetoplacental weight ratios were also calculated, which has been used as a measure of placental insufficiency.^30^ Statistical significance was detected for acute and fractioned treatment groups compared to sham-irradiated controls for the weight ratio (Figure 5). Based on the weight of the fetus and placenta individually, the decreased fetal weight was likely driving the significant decrease in the fetoplacental ratio.

## Discussion

The goal of this study was to characterize radiation-induced growth restriction during prenatal development following late gestational X-ray irradiation in a mouse model. Intrauterine growth restriction can be caused by maternal stress, which is why efforts were taken to minimize handling stress to the pregnant animals. Animals were acclimated to routine handling by the investigators prior to and following breeding. The Control and sham irradiation treatment groups were compared to assess any impacts of the sham irradiation protocol on the observed outcomes. Sham-irradiated animals were transported to the irradiator daily, to simulate the irradiation conditions. A previous study by our group had demonstrated transportation effects in a different prenatal mouse irradiation model.^4^ This was based on significant growth and cardiovascular outcomes between the sham and control treatment groups in that study. A distinguishing feature between Sreetharan et al. (2019)^4^ and the current study was the location of the animal irradiator. Sreetharan et al. (2019) required transportation of pregnant animals externally to a different building in a temperature-controlled vehicle. This is in contrast to this study, where the irradiator was located within the same building and animal facility. The lack of significant differences between the sham and Control groups in the present study suggests that the observed growth restriction responses are due to the radiation treatment, rather than the presence of confounding maternal stress caused by transportation or other aspects of the irradiation protocol. The only statistically significant study measure between the Control and sham-irradiation groups was placental weight. It should be noted that the significant decrease was small (0.011 ± 0.2 g in Control compared to 0.096 ± 0.01 g) and the standard deviation of this parameter was relatively much smaller compared to other endpoints (Table 3). This, in combination with the lack of fetal growth restriction between the two treatment groups would suggest that this statistically significant difference between the placental weights was not biologically significant.

A total of 5 irradiation conditions were tested during late gestation in this study, ranging from gestational day 14 to day 17. All irradiated dams demonstrated a reduced gestational weight gain post-irradiation, as shown in Figure 2. Data are presented for individual dams, which illustrates that the reduction in maternal weight gain was consistent across the treatment group, and not biased by an abnormally large or small outlier female. Evidence for radiation induced growth restriction was observed in the 1.82 Gy (day 14), 1.82 Gy (day 15), 61 mGy +1.82 Gy (day 14 + 15) and fractionation (0.455 Gy x 4, days 14-17) groups, based on a significant decrease in a combination of fetal weight or size measurements (Figure 3). Fractionation of the 1.82 Gy over four consecutive days (0.455 Gy x 4 group) resulted in a relatively equivalent level of growth restriction compared to an acute dose of 1.82 Gy. Although the outcome is highly context dependent, generally dose fractionation results in attenuated cellular responses compared to the same dose delivered in a single treatment. This is generally thought to occur due to the period between fractions during which the cell and system is capable of repairing damage caused by the irradiation, re-assortment of the cell population in the cell cycle and repopulation of cells from cell divisions.^31^ Other studies have also reported significant growth restriction in murine (mouse and rat) models following fractionated irradiation during prenatal development. Weber & Schmahl (1979)^32^ irradiated NMRI mice between gestational days 11-13 with 3 × 1.05 Gy fractions of 180 kV x-rays. The authors reported significant decreases in both fetal and placental weight with irradiation. Another study by Vidal-Pergola et al. (1993)^33^ tested the effects of ^60^Co gamma irradiation in Sprague-Dawley rats on gestational day 15, with either 0.5 Gy, 1.0 Gy or 2 × 0.5 Gy (delivered 6 h between fractions). The authors reported a significant dose-dependent decrease in offspring body weight, with minimal difference between the 1.0 Gy dose delivered as a single acute dose or fractionated into two fractions. This would suggest that the fractionated daily dose of 0.455 Gy was still large enough to induce growth restriction changes and possibly changing the fractionation schedule by modifying the dose per fraction or time between fractions could be interesting modifications for future work. Surprisingly, the 1.82 Gy (day 16) treatment group did not show evidence for growth restriction in any of tested fetal measurements. This may have been due to the timing of terminal sample collection in the study design, with dams being euthanized on gestational day 18, which is only 48 hours following 1.82 Gy radiation exposure. This could indicate that the growth restriction phenotype may require more time to manifest at the fetal level. This does not preclude the possibility that there are no other effects that could predispose the offspring to adverse health outcomes later in life after birth. Future studies that test later timepoints, such as birth weight or measurements later in postnatal life could provide evidence to verify this hypothesis.

The placental weight was not as responsive to radiation treatment, as there were no statistically significant differences detected between any of the treatment groups relative to sham-irradiated controls (Figure 4A). Philippe (1975)^34^ and Ward et al. (1971)^35^ reported decreased size of the murine placenta following acute prenatal irradiation; however, the tested doses in these studies were higher than the present study. Philippe et al. (1975) reported significant placental effects at the higher dose of 4.5 Gy on GD 10 and 11, and Ward et al. (1971) reported a significantly decreased placental wet weight at 300 and 500 Roentgen at an earlier timepoint of GD 5. A study by Kanter et al. (2014)^36^ reported a significant decrease in placental (and fetal) weight, following a 4 Gy ^137^Cs gamma irradiation on GD 14. Finally, a study by Schmahl (1979),^37^ which irradiated mice on GD 12 with 2 Gy of x-rays (similar to the dosing of the present study) did not observe significant differences in placental weight. These studies collectively would suggest that the threshold radiation dose for growth restriction of the placenta is likely greater than 2 Gy for photon irradiation. The observed significant differences fetoplacental weight ratio between treatment groups (Figure 4B) were therefore likely a consequence of the decreased fetal weight component of the ratio, coupled with an unchanged placental weight component.

One group in the present study was irradiated with a combination priming dose treatment of 61 mGy on day 14, followed 24 h later by a challenge dose treatment of 1.82 Gy on day 15. This group in combination with the 1.82 (day 15) group were used to test for the possibility of an adaptive response (AR). In this study, we did not observe evidence for an AR with a 61 mGy priming dose, as the fetoplacental measurements in the (61 mGy +1.82 Gy) combination group was unchanged compared to the group that only received the 1.82 Gy challenge dose on the same day of gestation (day 15). Despite the lack of observed effects on fetoplacental growth, there is the possibility that other cellular and genetic changes could have occurred, which would require further testing. It is difficult to determine exactly why an AR was not observed in this study, as there are a number of experimental conditions that could be responsible including priming or challenge absorbed dose, dose-rate, gestational timing for the treatments or timing between the priming and challenge dose. Modifying the priming dose used could be one option.

When considering the prenatal AR literature in mouse and rat models (Table 1), it is possible that the priming dose in this study was not large enough to induce an AR. This is based on previous reports in studies such as Boreham et al. (2006)^11^ that demonstrated an AR with a treatment of 0.3 + 4 Gy of ^60^Co gamma radiation in the gestational day 10-12 range. At the same time, a different report by Lee et al. (2008)^18^ did not observe evidence for an AR with a 0.3 + 0.8 Gy irradiation treatment in ICR mice, which suggests that it is not guaranteed that the use of a larger magnitude priming treatment (such as 0.3 Gy instead of 0.062 Gy as performed) would induce an AR in the study model from this study. The choice of the 61 mGy priming dose in the present study was selected based on a previous unpublished study from our group, and the previously mentioned Boreham et al. (2006) study. The chosen challenge dose in the current study (2 Gy nominal dose) was only half of the 4 Gy challenge dose in the Boreham et al. study, which is why the priming dose was scaled down to a nominal dose of 100 mGy. Following dosimetry verification, the actual absorbed dose was closer to 61 mGy. This was likely due to attenuation of the low energy x-rays, as the X-ray irradiator produces x-rays ranging from 0 to 320 kV. As discussed however, it is not guaranteed that an increase in priming dose alone would have resulted in an observed AR.

In addition to the choice of priming dose, there are other factors that could influence the AR, and could be variables that could be manipulated in the future to demonstrate AR. This includes the magnitude of the challenge dose. In the present study, the challenge dose of 1.82 Gy demonstrated robust growth restriction, and therefore appears to be appropriate for this mouse strain. The late gestational timing of GD 14 is one possibility. A study by Hays et al. (1993)^15^ demonstrated an AR in Sprague-Dawley rats at GD 14 also demonstrated evidence for an AR, however the timing between irradiations was on the scale of hours, rather than a full day. Most other rodent studies summarized in Table 1 tested the AR at earlier, more mid-gestational time points which could be one consideration for why we did not observe an AR. The dose-rates used in the present study are another variable for consideration. When comparing the dose-rates in the present study to other published AR studies in Table 1, the dose-rates in the present study appear to be within the same range of other published reports. It is important to note that apart from dose-rate, the energy of the X-ray photons is another consideration. For example, external irradiations with an isotope such as ^137^Cs results in gamma photons of a discrete energy (662 kV), whereas the X-ray irradiator in the current study would have exposed animals to a spectrum of energies ranging from 0 to 320 kV.

The present study has contributed towards a rodent model of radiation-induced growth restriction, although there were limitations to the present study that should be considered. The method of sample size selection (number of dams per irradiation treatment group) was based on previous experiments from our group, and a more robust power analysis to calculate the treatment group sizes could represent an improvement in future research. Another limitation was the testing of only a single AR treatment scenario. As discussed, there are a large number of factors that could influence the AR, and future studies that test further variations (example testing the AR at multiple gestational time points) could better characterize the AR in this study model. Finally, another important limitation was the choice to euthanize animals after a limited time period following irradiation. Future work that allows animals to develop postnatally will allow for the collection of critical measures such as birth weight or early life weight gain up to weaning are important metrics that were not possible in the present study due to this choice of sample collection timing. Further, other relevant endpoints that could be studied postnatally (ex. Generation of F2 offspring or other physiological endpoints later in life) would be relevant endpoints for future research if animals were allowed to continue past gestational development.

## Conclusion

The present study demonstrated growth restriction in BALB/c mice following high dose prenatal X-ray irradiation during late gestation, ranging from GD 14-17. The selected dose of 1.82 Gy demonstrated intrauterine growth restriction when irradiation was performed on GD 14, 15 and 16, with the greatest degree of growth restriction on GD 14. Fractionation of the dose over a total of 4 days (GD 14-17) also resulted in a significant growth reduction. Growth restriction of the offspring was correlated with a stunted gestational weight gain in the irradiated dams. The main objective of the present study was the development of an X-ray radiation-induced growth restriction model, and this goal was achieved in a number of irradiation conditions.

This model will be valuable for developmental biology and clinical researchers, and further molecular analysis of growth restricted fetuses and placenta samples represent avenues for future research. For example, DNA damage or oxidative stress markers could be further analyzed in collected tissues. In addition, the development of this model will allow for future studies with pre-treatment of animals with interventions (such as an antioxidant compounds). This could represent an opportunity to develop better countermeasures that are able to protect against the harmful effects of radiation exposure during development such as the ones observed in this study. Finally, there is also the possibility to extend this research in the future by monitoring physiological outcomes following birth of the offspring, potentially even later in life. Performing experiments on physiological outcomes that have been previously reported to be altered following prenatal exposure to high doses of ionizing radiation could include cardiovascular endpoints, neurobehavioural changes (by performing both behavioural testing and quantifying corresponding changes in brain tissue), growth trajectory of offspring into adulthood or generating F2 generation animals to understand if these effects can be transmitted to future generations all represent areas of novel research that can be extended from the work of the present study.
